# Remote sensing inversion of nitrogen content in silage maize plants based on feature selection

**DOI:** 10.3389/fpls.2025.1554842

**Published:** 2025-03-06

**Authors:** Kejing Cheng, Jixuan Yan, Guang Li, Weiwei Ma, Zichen Guo, Wenning Wang, Haolin Li, Qihong Da, Xuchun Li, Yadong Yao

**Affiliations:** ^1^ College of Water Conservancy and Hydropower Engineering, Gansu Agricultural University, Lanzhou, China; ^2^ State Key Laboratory of Crop Science in Arid Habitat Co-constructed by Province and Ministry, Gansu Agricultural University, Lanzhou, China; ^3^ College of Forestry, Gansu Agricultural University, Lanzhou, China; ^4^ College of Environmental Science and Engineering, Beijing University of Technology, Beijing, China

**Keywords:** vegetation indices, multispectral, unmanned aerial vehicle (UAV), feature importance scores, machine learning

## Abstract

Excessive nitrogen application and low nitrogen use efficiency have been major issues in China’s agricultural development, posing significant challenges for field management. Nitrogen is a critical nutrient for crop growth, playing an indispensable role in crop development, yield formation, and quality enhancement. Therefore, precisely controlling nitrogen application rates can reduce environmental pollution caused by excessive fertilization and improve nitrogen use efficiency. This study employs multispectral remote sensing images, combined with field-measured nitrogen content, to develop canopy nitrogen content inversion models for maize using three algorithms: backpropagation neural network (BP), support vector machine (SVM), and partial least squares regression (PLSR). The results reveal that there is a degree of redundancy in the information contained in various spectral indices. Feature selection effectively eliminates correlated and redundant spectral information, thereby improving modeling efficiency. The spectral indices Green Index (GI) and Nitrogen Reflectance Index (NRI) exhibit strong correlations with nitrogen content in the maize canopy, suggesting that the green and red spectral bands are crucial for retrieving maize’s biophysical and biochemical parameters. In studies on nitrogen content inversion in the maize canopy, the random forest (RF) algorithm, coupled with PLSR, demonstrated superior predictive performance. Compared to the standalone PLSR model, accuracy improved by 3.5%–6.5%, providing a scientific foundation and technical support for precise nitrogen diagnosis and fertilizer management in maize cultivation.

## 1 Introduction

Nitrogen is an essential component of proteins, chlorophyll, and many enzymes in plants. It is one of the indispensable nutrients for crop growth and development, and also serves as an important indicator for monitoring crop growth status (Du et al., 2023). The content levels of nitrogen directly influence crop yield and quality, both of which are essential for national food security and the sustainable development of agriculture. Traditional methods for assessing crop nitrogen content primarily involve destructive sampling and laboratory analysis, such as the Kjeldahl method, combustion method, and distillation method. These techniques are time-consuming, labor-intensive, and inefficient, making large-scale, non-destructive diagnostics infeasible and hindering the advancement of large-scale agriculture (Bao et al., 2024). Therefore, efficiently and rapidly monitoring crop nitrogen content is of significant importance for achieving precision agriculture and modern agricultural production (Yin et al., 2022).

In recent years, research on UAV-based remote sensing technology for crop monitoring has gained significant attention and has become one of the most crucial areas of study in agriculture (Dong et al., 2024). In the past, satellite remote sensing technology provided some data support for monitoring crop growth; however, its revisit cycle limitations and low resolution when collecting small to medium-scale agricultural field information hindered its ability to meet the demands of precise crop growth monitoring at the field scale (Sishodia et al., 2020). UAV remote sensing technology, with its advantages of low cost, high resolution, and strong timeliness, effectively compensates for the limitations of satellite remote sensing and has been widely applied in crop nitrogen nutrition diagnosis and monitoring (Liang et al., 2023). Currently, research on UAV-based remote sensing technology for crop monitoring mainly focuses on the remote sensing inversion of parameters such as crop water content, nitrogen content, and biomass (Alckmin et al., 2022; Shendryk et al., 2020; Shu et al., 2022). Wan et al. (2020) utilized UAV-acquired RGB and multispectral images of rice canopy to develop a model for predicting moisture content. The results indicated that the integration of vegetation indices and texture features significantly improved the accuracy of moisture content prediction. Wei et al., (2019) employed maize image data, in combination with field-measured leaf nitrogen content, to analyze the correlation between spectral variables and nitrogen content. The study found strong correlations between the green band index (GRE), the normalized difference vegetation index (GNDVI), and leaf nitrogen content. Wang et al. (2024) investigated methods for estimating rice biomass using UAV multispectral images and proposed a biomass estimation model based on vegetation indices. These studies provide effective management models and scientific guidance for precision agriculture, rational resource allocation, and the improvement of both yield and quality.

While UAV-based remote sensing technology has achieved significant results in crop monitoring, feature selection has become a critical step to further enhance the accuracy of remote sensing inversion. It plays an indispensable role in realizing high-quality remote sensing applications. Zhao et al. (2021) proposed a method for multi-source remote sensing soil moisture inversion in agricultural fields, combining feature selection and genetic algorithm optimization with a BP neural network. By performing proper feature selection, it is possible to identify features that are highly correlated with the inversion target, possess significant distinguishability, and exhibit stability (Han et al., 2023). This approach effectively reduces data redundancy and noise interference, lowers computational complexity, and enhances both the efficiency and accuracy of the inversion model, thereby improving its interpretability and predictive capabilities (Zhou et al., 2021). Existing studies have not sufficiently refined the handling of data quality and noise during feature selection. Low-quality data or inadequately processed noise can mislead feature selection, thereby reducing the accuracy of remote sensing inversion (Lan et al., 2024). Mao et al. (2022) proposed a non-parametric feature selection algorithm that combines random forests and deep neural networks. Through theoretical analysis and experimental validation, the advantages of this algorithm in identifying useful features, avoiding irrelevant features, and improving computational efficiency were demonstrated. RF are capable of handling high-dimensional and large-scale remote sensing data. By evaluating feature importance, they can accurately identify key features, reduce data redundancy, and minimize noise. Moreover, random forests can automatically capture nonlinear relationships between features, overcoming the traditional reliance on linear relationships. This makes them a more effective solution for complex remote sensing inversion problems (Sun and Chai, 2023; Zhou et al., 2016).

In this study, silage maize is used as the research subject. Multispectral UAV imagery of maize canopy at different growth stages is acquired, and supervised classification using ENVI 5.3 software is applied to eliminate soil background and shadows, resulting in the maize canopy spectral reflectance. Seventeen common vegetation indices (VIs) are established, and sensitive spectral indices are selected using a RF model. BP, SVM, and PLSR models are constructed to explore the optimal prediction model for silage maize at different growth stages. Based on the best model, a spatial distribution map of nitrogen content in the maize canopy is inverted, aiming to provide a fast and non-destructive technique for monitoring nitrogen content in field maize at different growth stages.

## 2 Materials and methods

### 2.1 Overview of the study area

**Figure 1:**
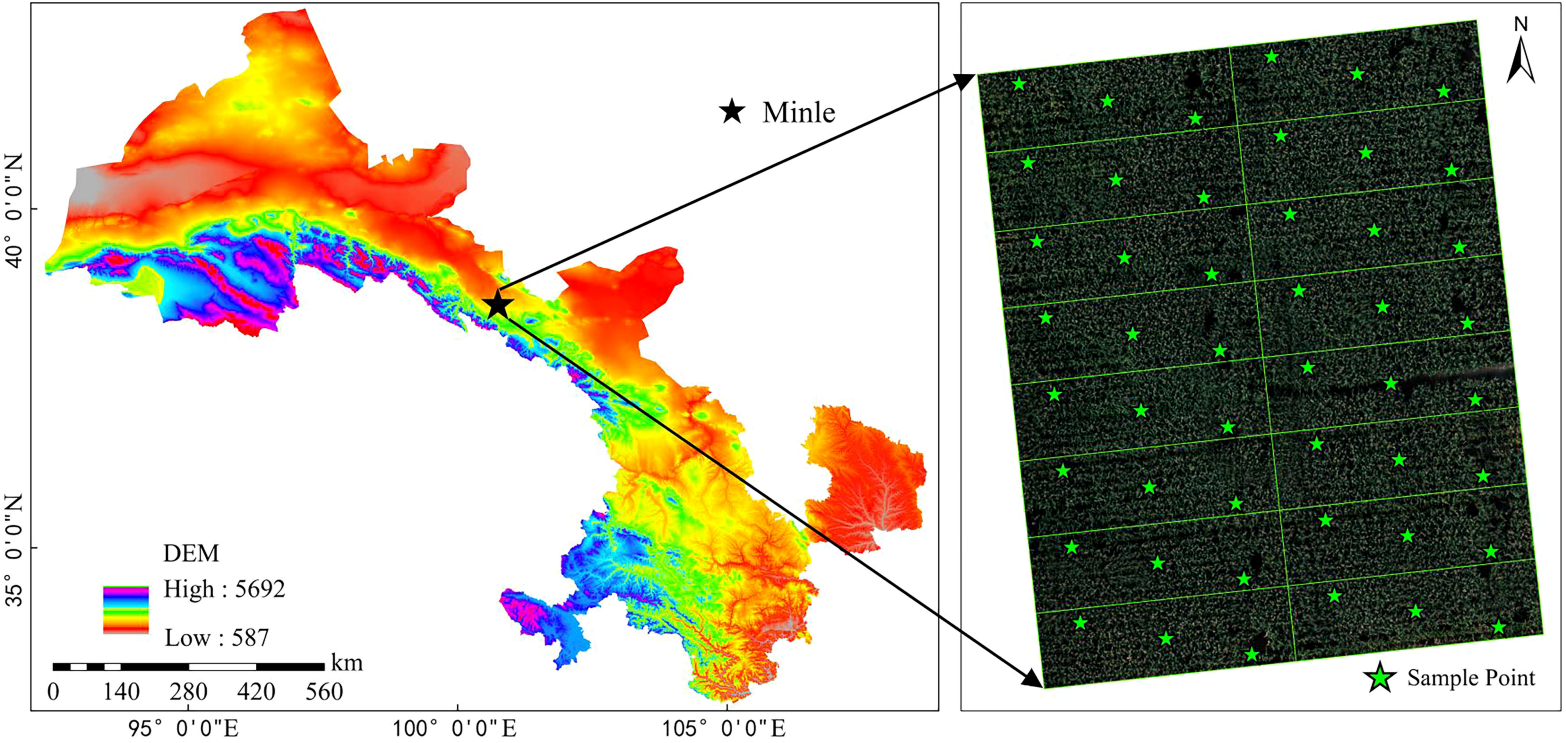
Overview of the study area.

This experiment was conducted at Huarui Farm, located in Minle County, Zhangye City, Gansu Province. Minle County lies in the central part of the Hexi Corridor, in the southeastern region of Zhangye City, with geographical coordinates ranging from 100°22′59″E to 101°13′9″E and from 37°56′19″N to 38°48′17″N. The county extends 73.8 km east to west and 95.4 km north to south, covering a total area of 3,687.32 km². Minle County experiences a continental desert steppe climate, characterized by prolonged sunshine, abundant thermal resources, large temperature fluctuations, and low precipitation. The average annual precipitation is 351 mm, while the average annual temperature is 4.1°C, with a frost-free period of approximately 154 days. Major rivers in the region include the Minle, Heihe, and Malian rivers. The elevation ranges from 1,589 m to 5,027 m from north to south. The dominant soil types include aeolian sand, gray-brown desert soil, gray calcareous soil, and millet calcareous soil. The study area is shown in **Figure 1**, and the experimental plot was divided into 16 subplots. A diagonal sampling method was employed for uniform sampling, with a total of 48 samples.

### 2.2 Data acquisition and preprocessing

#### 2.2.1 Acquisition and processing of multispectral data

Multispectral UAV imaging was conducted on maize at four key growth stages: the seedling stage (May 18), jointing stage (July 1), tasseling stage (August 4), and milking stage (September 10). To ensure the accuracy and reliability of the multispectral data, observations were performed under clear weather conditions with minimal wind disturbance. Data collection occurred between 11:00 AM and 1:00 PM, and UAV remote sensing data were obtained prior to plant sampling to confirm that the plants sampled were captured in the multispectral images. The DJI M300 RTK UAV, equipped with the MS600 Pro multispectral camera, has a maximum flight time of 55 minutes and a transmission range of up to 15 kilometers. It is capable of carrying three payloads simultaneously, with a maximum load capacity of 2.7 kg. The multispectral sensor features six bands: blue, green, red, red edge 1, red edge 2, and near-infrared. The forward overlap was set to 75%, and the side overlap to 60%. The UAV flew at an altitude of 30 meters, with an image spatial resolution of 0.02 meters. The flight missions, each lasting approximately 30 minutes, were accompanied by synchronous ground-based imaging of calibration panels before and after each flight for reflectance calibration purposes. The deployment of calibration panels effectively compensated for illumination variations caused by changes in solar zenith angles and atmospheric conditions, thereby ensuring data consistency across different times within a single day and over multiple days. The band parameters of the multispectral camera are listed in **Table 1**.

**Table 1:** Band parameters of multispectral sensor.

Band	Band center/nm	Band width/nm
NIR	840	35
REG-2	750	15
REG-1	720	10
RED	660	20
GREEN	555	25
BLUE	450	35

**Figure 2:**
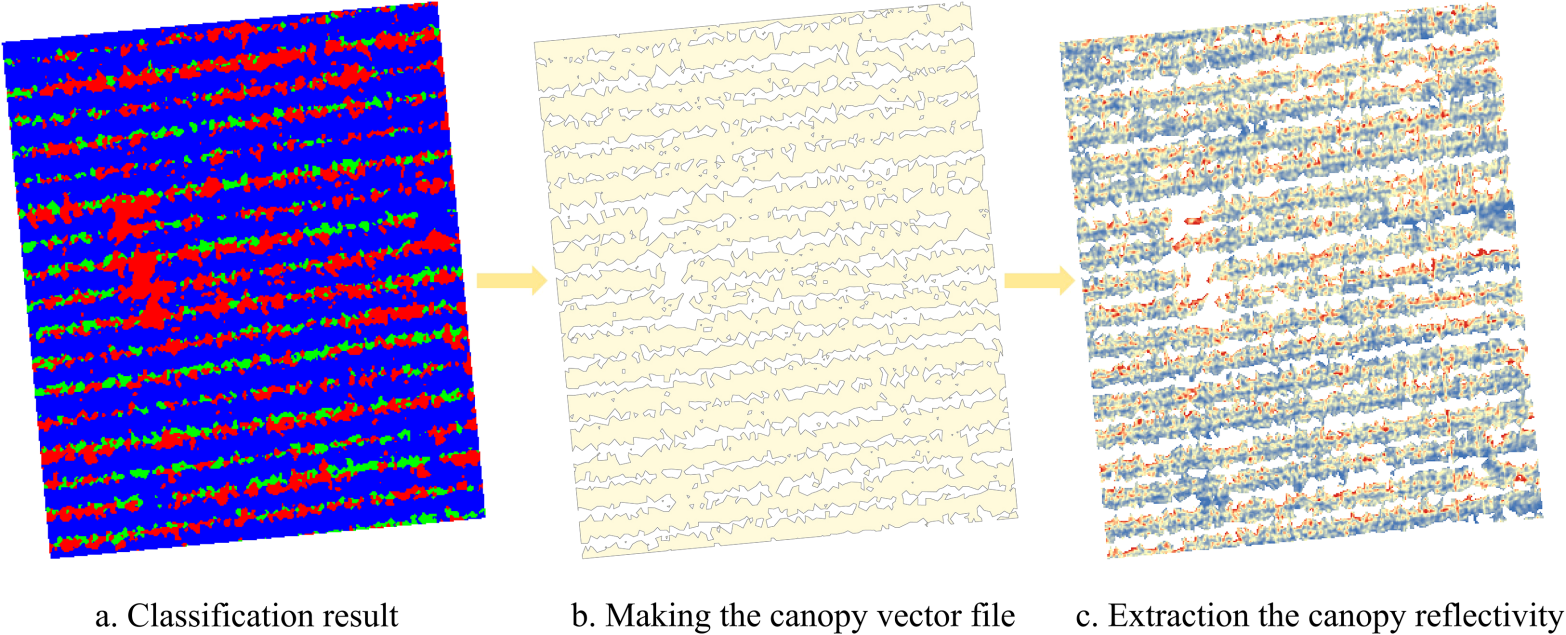
Extraction of canopy spectral reflectance. **(a)** Classification result. **(b)** Making the canopy vector file. **(c)** Extraction the canopy reflectivity.

To obtain a complete set of images for the study area, Pix4D Mapper software was used to stitch images acquired at different growth stages. Automatic aerial triangulation technology was employed to precisely georeference the images and compute stitching parameters, facilitating the construction of a high-precision point cloud model. Based on this, the spatial positions and stitching parameters of the original images were automatically optimized and calibrated, ultimately generating a high-resolution Digital Orthophoto Map (DOM) covering the entire experimental area. False-color composite images were generated using ENVI software, and supervised classification was performed to remove soil backgrounds and shadows. Finally, ArcGIS software was applied for mask clipping to extract reflectance values of each spectral band during different growth stages, which were subsequently used for VIs calculations (**Figure 2**).

#### 2.2.2 Statistical analysis of plant nitrogen content (PNC)

To minimize the potential influence of surrounding bare soil or adjacent fields, sampling should be conducted at least 0.5 meters from the edges of the experimental plots, ensuring a buffer zone from the field ridges. In each experimental plot, three maize plants exhibiting uniform growth and intact canopies were selected as samples. The coordinates of the sampling points were recorded using RTK. After removing the roots, the samples were separated into three categories: stems, leaves, and ears. These samples were sterilized in an oven at 105°C for 30 minutes and then dried at 85°C until they reached a constant weight. The dry weight was measured, and the three types of dried samples were ground into a fine powder and thoroughly homogenized. Following digestion with an H_2_SO_4_-H_2_O_2_ solution, the PNC was determined using a Kjeldahl nitrogen analyzer.

Mathematical statistical analysis of nitrogen content in plant samples from the study area was conducted. The maximum nitrogen content in maize ranged from 3.01% to 18.72% throughout the growing season, showing a trend of first decreasing and then increasing. This trend may be related to the contribution of nitrogen to changes in crop growth. Descriptive statistics of nitrogen content in the maize canopy are shown in **Table 2**.

**Table 2:** Descriptive statistics for the Nitrogen content in maize plants at different growth.

Date	Samples	Range/%	Mean/%	Standard Deviation/%	Coefficient of Variation/%
Seedling	48	10.735~18.723	14.205	1.832	0.129
Jointing	48	5.489~8.150	6.349	0.501	0.079
Tasseling	48	3.012~4.210	3.632	0.270	0.074
Milking	48	12.340~16.892	14.461	1.006	0.070

**Figure 3:**
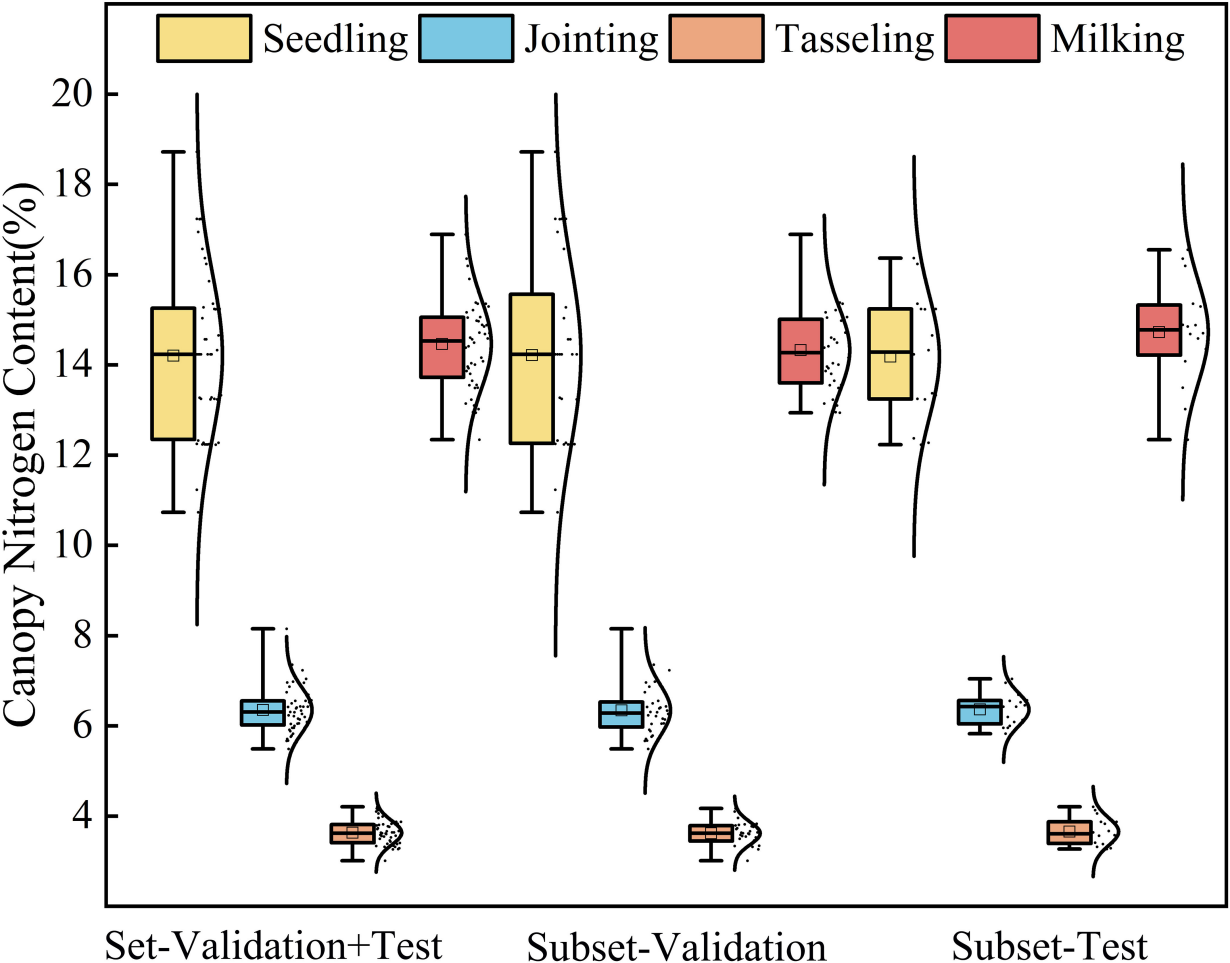
Box plot showing the statistical results of canopy nitrogen weight (g/m^2^).

The original dataset was sorted in ascending order and subsequently divided using systematic sampling at fixed intervals. Starting from the first element of the sorted dataset, every third sample was selected for the validation set (comprising 16 data groups), while the remaining samples formed the modeling set (consisting of 32 data groups). **Figure 3** shows the statistical results of nitrogen content in the maize canopy. It can be observed that both the training and validation sets for different growth stages maintained statistical results similar to the overall dataset, minimizing the bias between the training and validation sets.

### 2.3 Calculation of VIs

VIs are constructed by linear or nonlinear combinations of different spectral bands. VIs are designed to integrate various related spectral signals, enhancing information from vegetation while minimizing the effects of external factors such as soil background, climate, and vegetation cover variability (Qiao et al., 2022). The use of multispectral UAV remote sensing data to calculate VIs for each band has become the most commonly used method for monitoring PNC. Most researchers have employed VIs derived from the visible spectrum and selected those with a high correlation to ground truth data as input variables for model development, achieving satisfactory prediction results. This indicates that the establishment of VIs offers a distinct advantage in PNC monitoring applications (Shah et al., 2019).

Studies have shown a significant correlation between VIs, PNC of crop, and biomass. By linearly or non-linearly combining spectral reflectance from different characteristic bands, VIs can be used to invert various vegetation parameters and diagnose vegetation growth status (Ma et al., 2022). In this study, multispectral UAV data is combined with existing PNC inversion research to select six spectral band reflectances and 17 VIs for the inversion of nitrogen content in the maize canopy. The calculation formulas for the VIs are shown in **Table 3**, and these indices will be used as input variables for the nitrogen estimation model in subsequent analysis.

**Table 3:** Vegetation indices used in this study.

Vegetation Index	Name	Formula
DVI	Difference Vegetation Index	NIR−RED
GNDVI	Green Normalized Difference Vegetation Index	(NIR−GREEN)/(NIR+GREEN)
SAVI	Soil-Adjusted Vegetation Index	(NIR−RDE)*(1+0.5)/(NIR+RED+0.5)
BNDVI	Blue Normalized Difference Vegetation Index	(NIR−BLUE)/(NIR+BLUE)
NDVI	Normalized Difference Vegetation Index	(NIR−RED)/(NIR+RED)
RVI	Ratio Vegetation Index	NIR/RED
WDRVI	Wide Dynamic Range Vegetation Index	(0.12*NIR−RED)/(0.12*NIR+RED)
GDVI	Green Difference Vegetation Index	NIR−GREEN
ISR	Improved Soil-Adjusted Ratio	RED/NIR
NDREI	Normalized Difference Red Edge Index	(REG750−GREEN)/(REG750+GREEN)
GI	Green Index	GREEN/RED
NR	Normalized Red	RED/(RED+GREEN+NIR)
NG	Normalized Green	GREEN/(RED+GREEN+NIR)
NNIR	Normalized NIR index	NIR/(RED+GREEN+NIR)
OSAVI	Optimized Soil-Adjusted Vegetation Index	(NIR−RED)/(NIR+RED+0.16)
NRI	Nitrogen Reflectance Index	(GREEN−RED)/(GREEN+RED)
DVI	Difference Vegetation Index	NIR−RED

NIR, REG2, REG1, RED, GREEN, and BLUE represent the spectral reflectance of the multispectral camera at wavelengths of 840, 750, 720, 660, 555, and 450 nm, respectively.

### 2.4 Model construction and accuracy evaluation

The BP, SVM, and PLSR machine learning algorithms were individually applied to develop separate inversion models for nitrogen content in the maize canopy. All of these regression algorithms were implemented using MATLAB.

#### 2.4.1 RF model

To analyze the key spectral variables influencing nitrogen content in the maize canopy, a quantification study using RF was conducted. RF is an ensemble learning method composed of multiple regression trees (Pang et al., 2022). There is no correlation between the decision trees in the forest. The final output of the model is determined collectively by each tree in the forest. Unlike traditional decision trees, random forests do not require predefined weights for each attribute. Instead, they randomly select a subset of data as variables from the sample data and use their attribute values to predict new samples (Everingham et al., 2016). RF utilizes an iterative algorithm to select the best decision trees, which are then used in ensemble learning. During the splitting of each decision tree node, RF randomly selects a subset of variables from the entire set to identify the optimal features, thereby improving prediction accuracy. Additionally, RF exhibits strong tolerance to noise and outliers, making it less prone to overfitting (Sun and Chai, 2023).

#### 2.4.2 BP model

BP is a multilayer feedforward neural network trained using the error backpropagation algorithm, also known as the backpropagation network (Tang et al., 2022). This algorithm is based on multilayer neural networks and exhibits features such as fault tolerance, automatic adjustment, and self-learning. It also possesses strong nonlinear mapping capabilities, allowing it to handle complex nonlinear relationships between inputs and outputs. As a result, it is one of the most widely used neural network models today (Song et al., 2021). During the training process, the error between the actual and expected outputs at the output layer is calculated. This error signal is then propagated backward from the output layer to the hidden layers, and finally to the input layer. Throughout this process, the connection weights and biases between neurons in each layer are adjusted according to specific rules, enabling the network’s output to gradually approach the desired output (Wang et al., 2022).

#### 2.4.3 SVM model

SVM is a powerful and widely used machine learning algorithm. It offers distinct advantages in small-sample, nonlinear, and high-dimensional pattern recognition, and is relatively tolerant to noise in the data (Sawut et al., 2021). Due to its focus on minimizing structural risk rather than empirical risk, it demonstrates strong generalization ability and can achieve good performance even with a limited number of training samples (Wang et al., 2016). Moreover, the solution of SVM exhibits sparsity, meaning that most training samples do not affect the model, and only the support vectors play a role. This results in relatively high computational efficiency and lower storage requirements, which is why SVM is widely used in spectral analysis research (Piccialli and Sciandrone, 2022).

#### 2.4.4 PLSR model

PLSR is a statistical method that combines principal component analysis (PCA), canonical correlation analysis (CCA), and multiple linear regression (MLR). It is primarily used to address data analysis problems involving multicollinearity among variables and a limited number of sample points (Ezenarro et al., 2023). By minimizing the sum of squared errors, the optimal matching function for a set of data can be found, which can effectively address multicollinearity issues between parameters to some extent (Alexis et al., 2023). PLSR demonstrates good adaptability and stability when dealing with high-dimensional and small sample data. By extracting key component information, it reduces the data dimensionality and prevents overfitting (Shen et al., 2020). Furthermore, PLSR can simultaneously model multiple dependent variables, making it suitable for regression problems involving multiple response variables.

#### 2.4.5 Accuracy evaluation metrics

To evaluate the predictive capability and fitting accuracy of the inversion model, three performance metrics were used: the root mean square error (RMSE), coefficient of determination (R²), and mean absolute error (MAE). R² measures the degree of correlation between the predicted and observed values, while RMSE quantifies the deviation between them. MAE represents the average error between the predicted and observed values. Higher R² values, approaching 1, and smaller RMSE and MAE values indicate better model fitting accuracy and enhanced prediction performance. R², RMSE, and MAE are calculated using Equations 1–3, respectively.


(1) $$R^{2}=\frac{\sum_{i=1}^{n}{\left(Y_{i}-\bar{X}\right)}^{2}}{\sum_{i=1}^{n}{(X_{i}-\bar{X}}^{{)}^{2}}}$$

(2) $$RMSE=\sqrt{\frac{\sum_{i=1}^{n}{\left(Y_{i}-X_{i}\right)}^{2}}{n}}$$

(3) $$MAE=\frac{1}{n}\sum_{i=1}^{n}\left|X_{i}-Y_{i}\right|$$

Here, n represents the total number of samples, denotes the observed mean value of the samples, and represent the observed value and the predicted value of the i-th sample, respectively.

## 3 Results and analysis

### 3.1 Canopy spectral reflectance extraction

**Figure 4:**
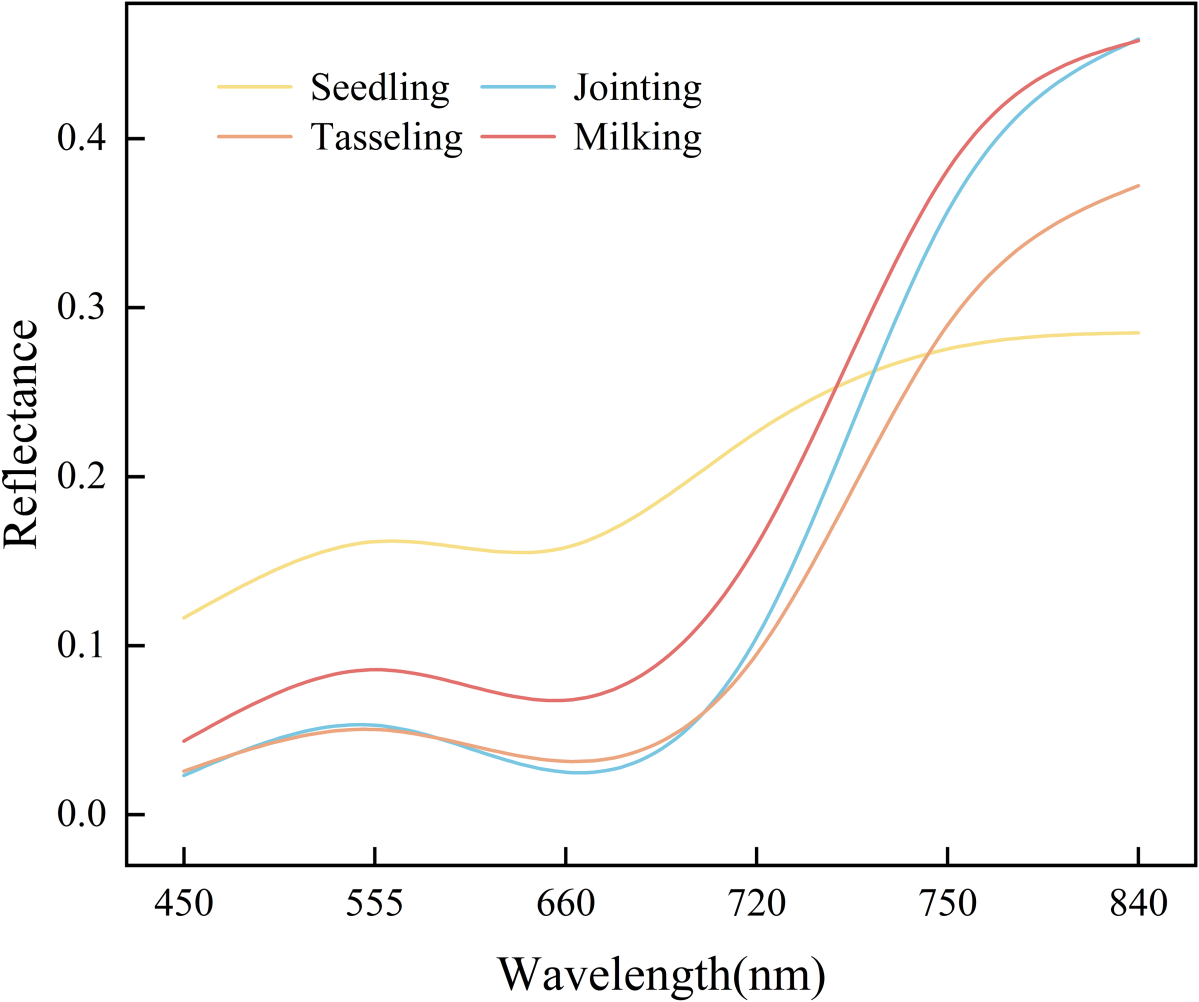
Spectral variation characteristics of maize canopy.

**Figure 4** illustrates the variation in spectral reflectance across the maize canopy at different growth stages. From the blue band (450 nm) to the near-infrared (NIR) band (840 nm), spectral reflectance follows a distinct pattern: initially increasing, then decreasing, and subsequently increasing again. A small peak appears in the green band (555 nm), while absorption troughs are observed in the blue and red bands (660 nm). In contrast, a sharp rise occurs, reaching a larger peak in the red edge (720 nm and 750 nm) and NIR bands. As the growing season progresses, the canopy reflectance exhibits significant dynamic changes. During the seedling stage, the canopy reflectance typically remains at a high level. As the plants enter the mid-growth stage, reflectance gradually decreases, reaching the lowest point of the entire growth cycle. In the milking stage, canopy reflectance increases again, recovering to a higher value as a result of changes in the plant’s physiological state.

### 3.2 Spectral index feature selection

The sensitivity of different spectral variables to PNC varies. A random forest algorithm was employed to analyze the correlations between six spectral bands and seventeen vegetation indices with plant nitrogen content. The contribution of each feature to the model’s prediction performance was assessed, and the importance of each feature was quantified, ultimately ranking the features based on their significance. Spectral variables with a feature weight greater than 4% were selected for each growth stage, and the results are presented in **Table 4**. At the seedling stage of maize, six features were selected by the random forest model, listed in descending order of importance: GI (22.2%), SAVI (19.5%), NRI (17.4%), OSAVI (7.7%), DVI (7.4%), and NG (4.2%). The ranking of feature variables at the jointing stage shows a decreasing trend in importance, from NDREI, NRI, GI, REG750, REG720, GDVI, RED, and SAVI to NG. NDREI contributed the most at 19.7%, while NG had the lowest contribution at just 4.2%. During the tasseling stage, the red light band was most sensitive to plant nitrogen content, followed by NRI. However, at the milking stage, the sensitivity of the red light band to plant nitrogen content decreased significantly, with a relatively low value of only 1.3%.

**Table 4:** Feature importance ranking.

Date	Input Variables	Number of Variables
Seedling	GI(22.2%)、SAVI(19.5%)、NRI(17.4%)、OSAVI(7.7%)、DVI(7.4%)、NG(4.2%)	6
Jointing	NDREI(19.7%)、NRI(11.8%)、GI(10.1%)、REG750(9.2%)、REG720(6.8%)、GDVI(6.5%)、RED(5.0%)、SAVI(4.3%)、NG(4.2%)	9
Tasseling	RED(19.4%)、NRI(18.2%)、GI(14.9%)、NR(11.4%)、NDREI(7.4%)、RVI(4.5%)	6
Milking	SAVI(20.0%)、OSAVI(17.6%)、GI(11.6%)、NRI(10.1%)、DVI(8.5%)	5

The correlation between nitrogen content and vegetation indices in maize plants exhibits significant variations across different growth stages, primarily attributable to the physiological characteristics of maize, dynamic changes in canopy structure, and temporal variations in spectral response features. During the seedling stage, the low canopy coverage and small leaf area index result in substantial interference from soil background on canopy spectral reflectance. Both GI and SAVI demonstrate superior resistance to soil background interference, effectively mitigating the impact of soil reflectance and thereby providing more accurate characterization of plant nitrogen status. The jointing stage, being a critical phase for maize vegetative growth, is characterized by canopy closure and a significant increase in leaf area index, accompanied by elevated levels of chlorophyll content and nitrogen demand. The NDREI demonstrates significant correlation with plant nitrogen content by utilizing red-edge bands that are sensitive to chlorophyll concentration changes, while the NRI is based on an optimized combination of red and green bands. During the tasseling stage, the maize canopy reaches full closure, with the leaf area index peaking at its maximum value for the growth cycle. At this stage, nitrogen demand is primarily concentrated in ear development and grain formation processes. Both RED and NRI exhibit high sensitivity in detecting the spatial distribution of nitrogen and vertical heterogeneity of chlorophyll content within the canopy, accurately reflecting the vertical distribution characteristics of plant nitrogen status. This provides reliable spectral evidence for nitrogen nutrition diagnosis during the tasseling stage. As the maize plants enter the senescence phase at milking stage, chlorophyll degradation leads to significant leaf yellowing, accompanied by a noticeable decline in leaf area index. Consequently, the influence of soil background on canopy spectral reflectance becomes prominent again. Both SAVI and OSAVI effectively mitigate soil reflectance interference on canopy spectra through the incorporation of soil adjustment factors, significantly enhancing the monitoring accuracy of plant nitrogen status.

**Figure 5:**
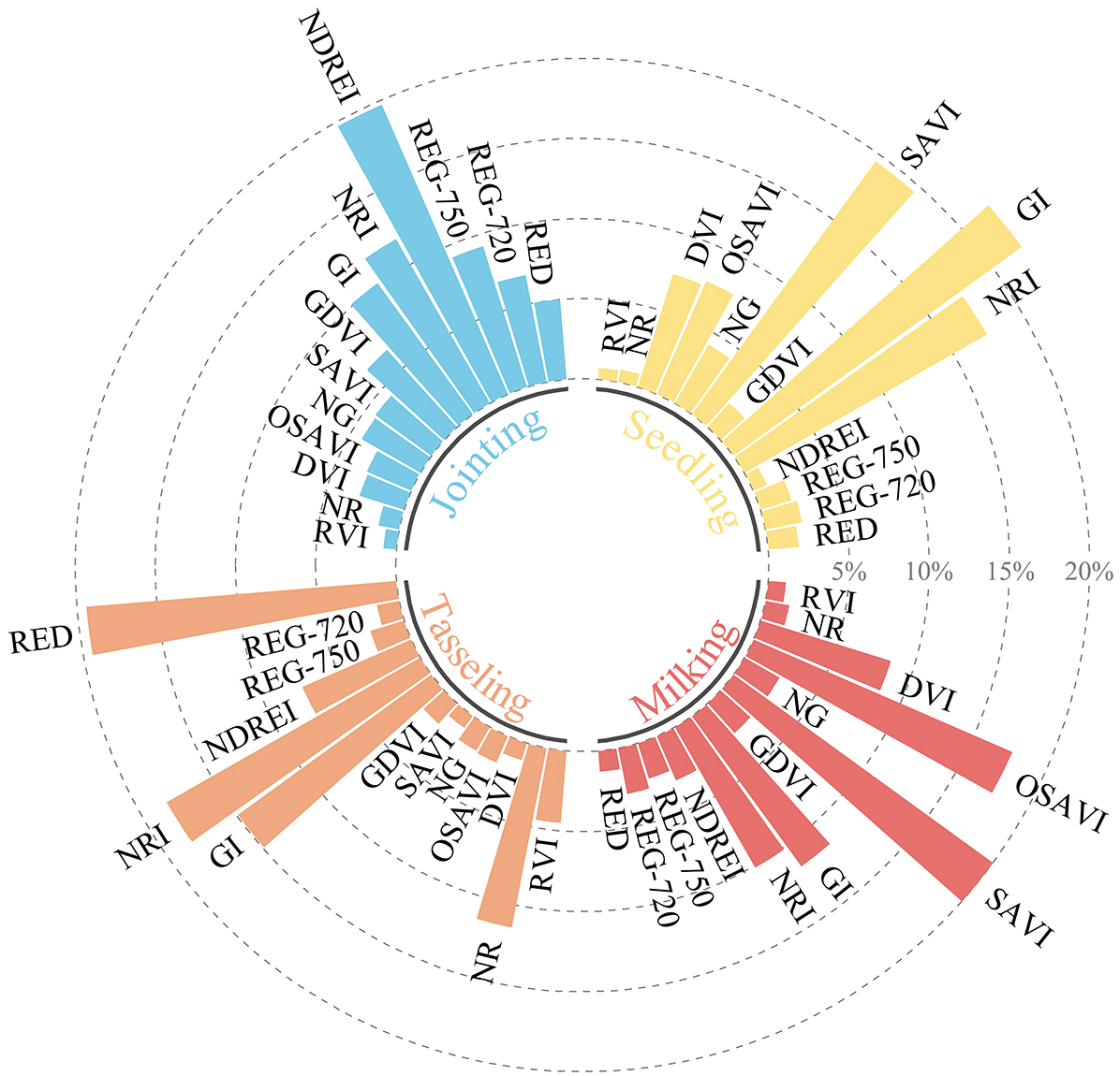
Proportion of feature importance.

**Figure 5** presents the proportion of key feature variables at different growth stages of maize when nitrogen content is inverted using various spectral indices. The importance of each variable radiating from the center increases incrementally by 5%. Both GI and NRI show a strong correlation with nitrogen content in the maize canopy, indicating that the red and green bands play a key role in the absorption and reflection of chlorophyll. Based on the correlation between red and green band reflectance and chlorophyll content, numerous studies have established various VIs to estimate PNC. When nitrogen content is sufficient, chlorophyll levels are relatively high, leading to increased absorption of red light and a decrease in green light reflectance. Therefore, the GI and NRI indices can indirectly reflect changes in nitrogen content within the leaves.

### 3.3 Comparative analysis of model accuracy

**Figure 6:**
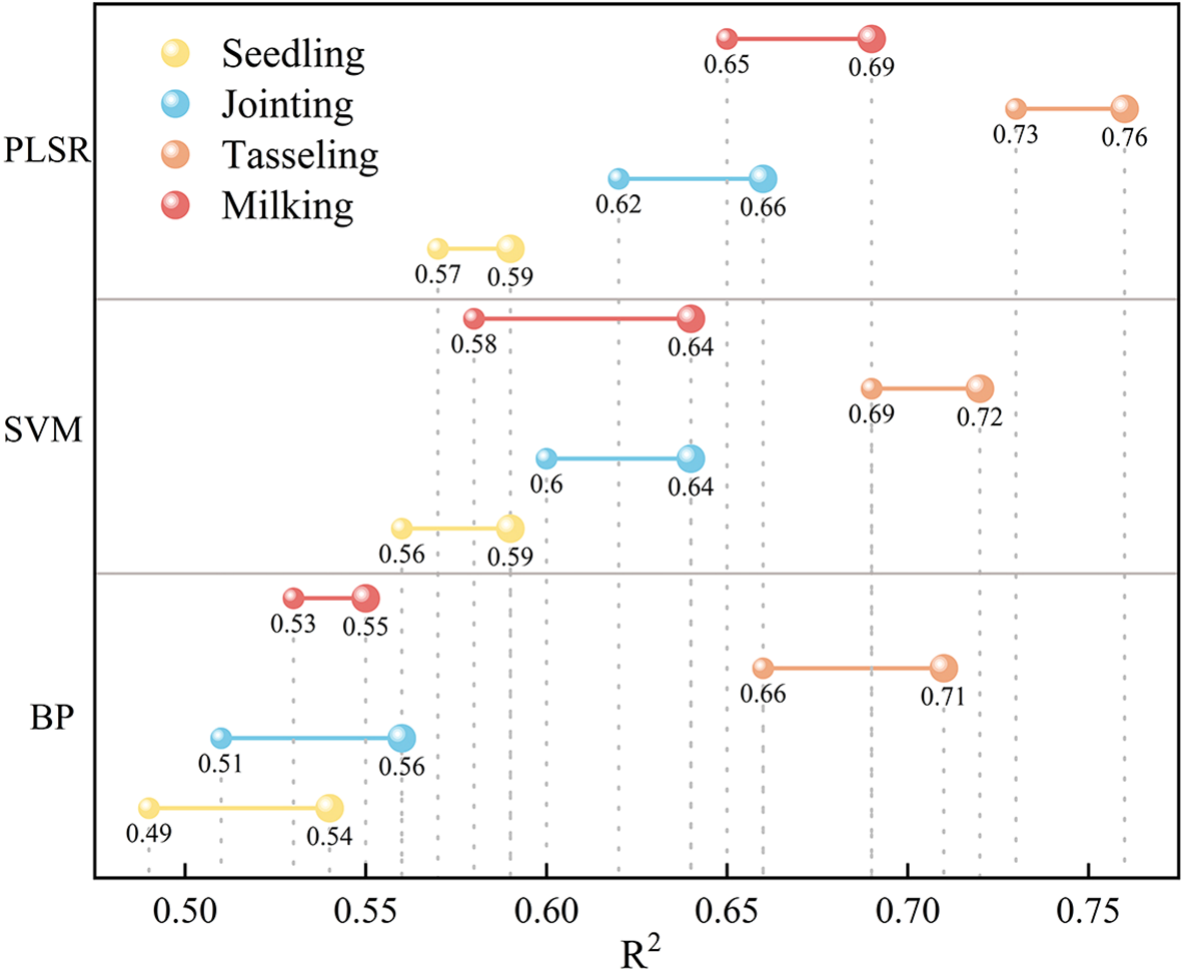
Comparison of model accuracy before and after feature selection.

Building on the previous analysis and the importance ranking of spectral variables in relation to maize canopy nitrogen content, three prediction models—BP, SVM, and PLSR—were developed using all spectral variables and the selected spectral variables as independent variables, with PNC as the dependent variable, for different growth stages. The results are presented in **Figure 6**.

Among all the models, PLSR achieved the highest prediction accuracy across all growth stages. After feature selection, the prediction accuracy of maize canopy nitrogen content improved to some extent at each growth stage. The comparative analysis of the impact of variable selection on prediction accuracy revealed that, after variable selection, the BP model’s estimation accuracy improved by 10.2%, 9.8%, 7.6%, and 3.8% for the seedling, jointing, tasseling, and milking stages of maize, respectively. The estimation accuracy of maize at milking improved by more than 10% using SVM, while the improvement in prediction accuracy with PLSR was generally lower before and after feature selection. This is because PLSR inherently has some feature selection capabilities. After feature selection, the prediction accuracy for all four growth stages was optimized to varying degrees, with the best performance observed during the jointing stage.

A comparative analysis of the performance of three modeling approaches reveals that the PLSR model provides the best predictive accuracy. When comparing the maize canopy nitrogen content inversion models developed using these methods, the model based on the PLSR algorithm achieved a maximum accuracy that was 5.56% higher than the SVM model and 7.04% higher than the BP model. Notably, the maize canopy nitrogen content inversion model constructed using RF feature selection combined with PLSR exhibited the best fit, with an R² of 0.76.

### 3.4 Spatial distribution of PNC

**Figure 7:**
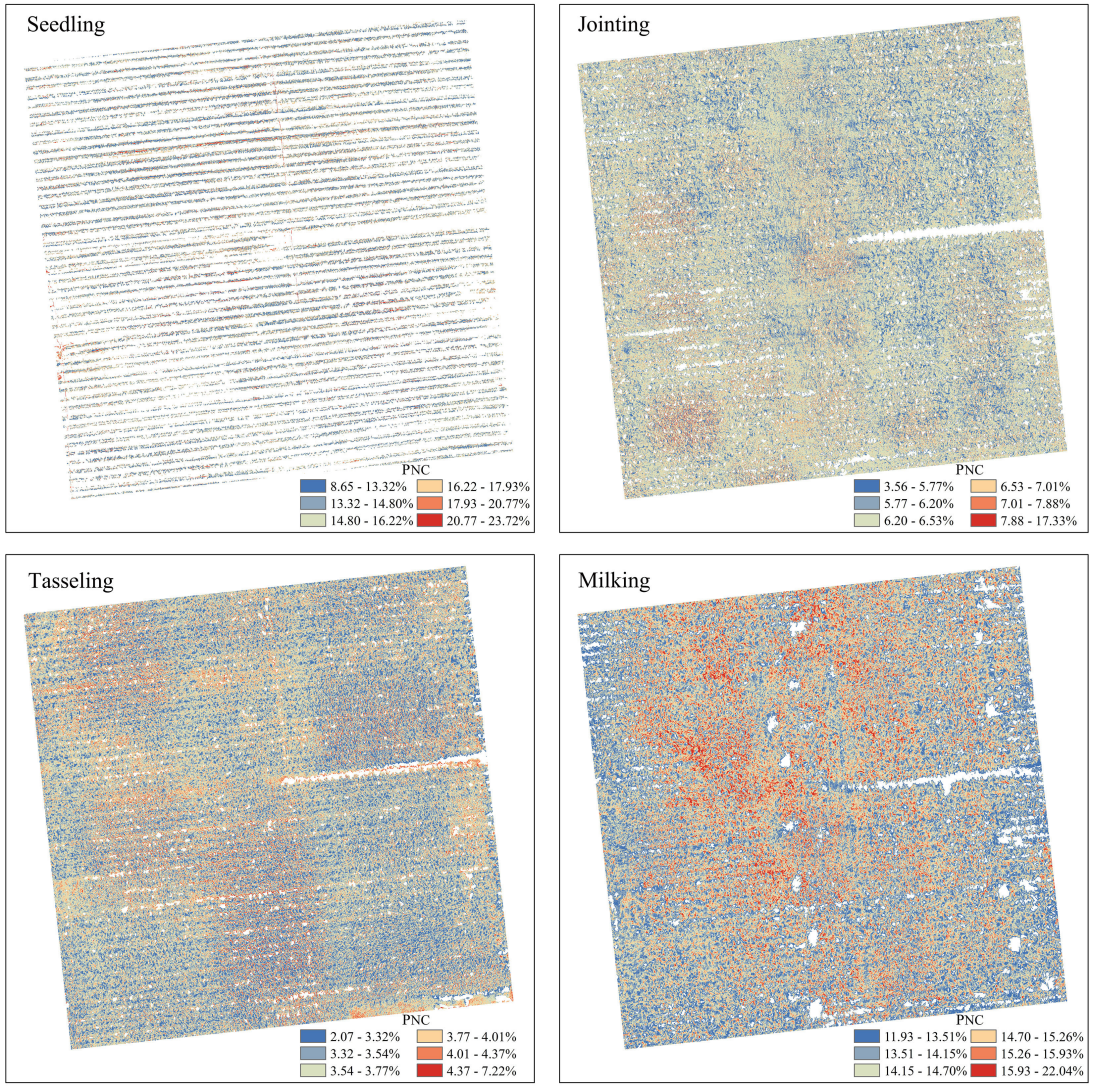
Spatial distribution map of PNC.

To improve the evaluation of the model’s inversion performance, a comparative analysis of the prediction results from three machine learning algorithms after feature selection is conducted. The optimal prediction model, RF coupled with PLSR, is utilized to estimate the nitrogen content in the maize canopy at various growth stages. The predicted values closely match the observed values, and the distribution of nitrogen content aligns well with the actual conditions. The results are presented in **Figure 7**.

## 4 Discussion

### 4.1 Inversion of PNC based on feature selection

Traditional approaches typically build crop nitrogen content estimation models based on a single spectral variable. However, models constructed using a single variable are prone to saturation, while using too many variables may lead to overfitting (Ma et al., 2024). Previous studies have shown that feature selection can effectively reduce redundancy in model input variables, thereby improving both model efficiency and accuracy. Lee et al (Lee et al., 2020). employed the random forest algorithm for feature selection, successfully identifying variables with minimal impact on model predictions. By removing redundant variables, they reduced the computational time of the model while simultaneously enhancing its prediction accuracy. The random forest importance plot can identify variables that have little or no impact on the model. Removing these variables not only reduces processing time but also improves prediction accuracy (Hua et al., 2023).

**Figure 8:**
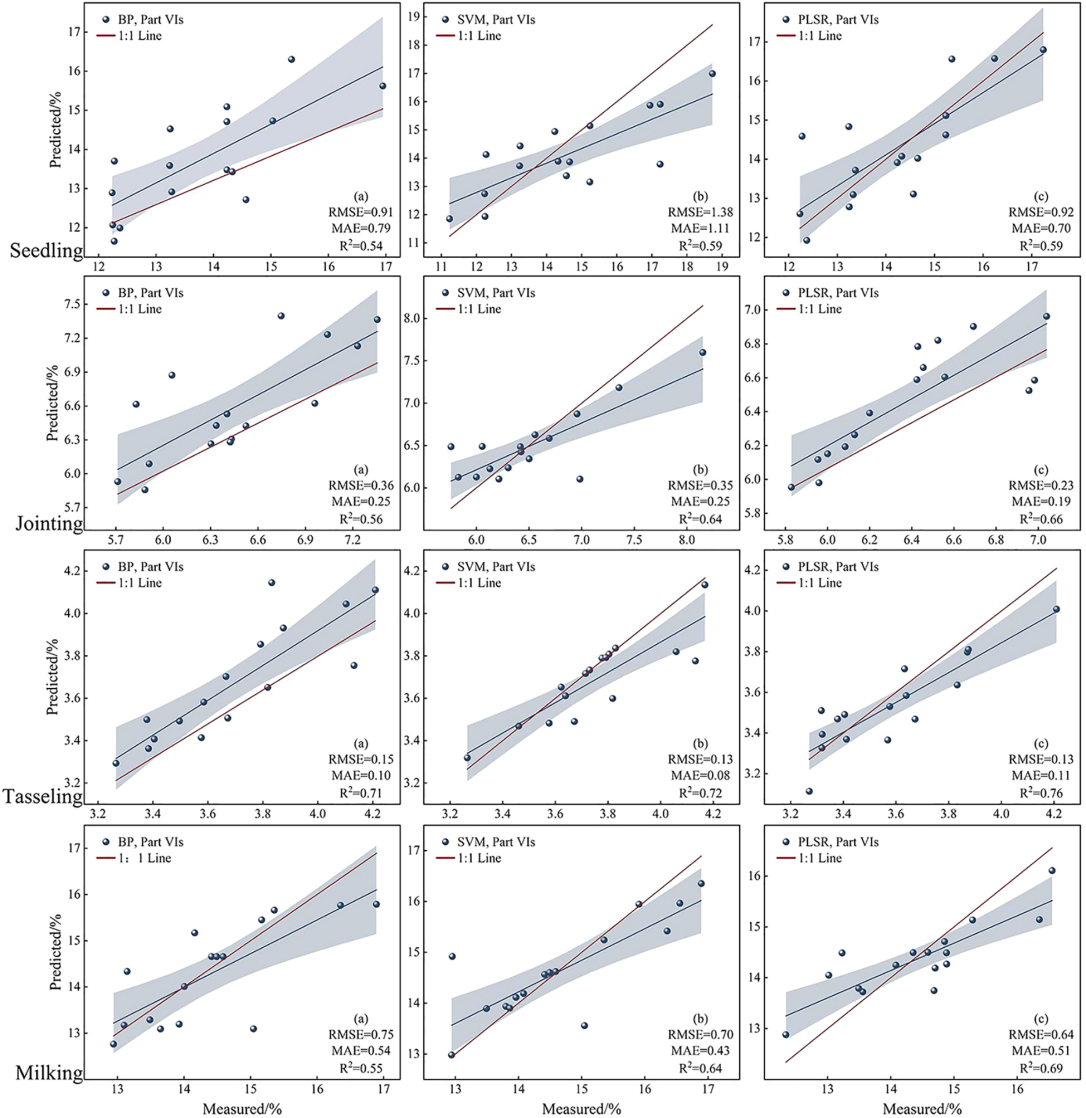
Linear fit of PNC predictions under different models.

As shown in **Figure 8**, the RF coupled PLSR model demonstrates better predictive performance in the maize canopy nitrogen content inversion, with an accuracy improvement of 3.5% to 6.5% compared to the single PLSR model. By comparing the prediction results of maize canopy nitrogen content at different growth stages, it was found that the highest prediction accuracy was achieved during the tasseling stage, which is consistent with the findings of Liu et al. (2012). This may be due to the relatively stable growth conditions of maize plants during this stage, where their physiological and morphological traits are more pronounced and representative, facilitating the extraction of relevant information associated with nitrogen content. Additionally, during the tasseling stage, external environmental factors such as temperature, light, and moisture are more stable, reducing the interference of environmental variables on nitrogen content prediction.

### 4.2 The impact of different models on the spatial distribution of PNC

The BP, SVM, and PLSR models were applied to estimate PNC. The results show that the BP model demonstrated strong nonlinear fitting capability, with an R² of 0.71 and an RMSE of 0.15. Alhnaity et al (Alhnaity et al., 2020). utilized deep learning techniques to predict plant growth and yield in greenhouse environments. The results indicated that the BP network performed exceptionally well in handling complex plant growth data. However, the training process is intricate and requires substantial computational resources and time. Additionally, the model is prone to overfitting, necessitating large amounts of training data to mitigate this issue. Furthermore, the choice of parameters has a significant impact on the results. The SVM model, with its advantages in handling small samples and high-dimensional data, yields relatively stable results, achieving an R² value of 0.72 and an RMSE of 0.13. Shahhosseini et al. (2019) evaluated the performance of five machine learning algorithms in predicting maize yield and nitrogen loss. The study found that SVM demonstrated better predictive capabilities under certain conditions, although its performance was dependent on the size of the training dataset and the selection of input variables. In studies of nitrogen content inversion in maize, it effectively handles noise and outliers in the data by finding an optimal hyperplane for classification or regression. When sample size is limited, the SVM model tends to be more stable than other models and can, to some extent, overcome overfitting. However, it is sensitive to the choice of kernel function and parameter tuning, requiring careful optimization to achieve optimal performance; The PLSR model has certain advantages in handling multivariate collinearity, with an R² value of 0.76 and an RMSE of 0.13. It simultaneously considers the relationships between multiple independent and dependent variables, effectively addressing the complex multivariate interactions. By performing a comprehensive analysis of the independent and dependent variables, the PLSR model can more fully reveal the underlying patterns in the data. Jiang et al. (2006) investigated the application of PLSR in short-term climate forecasting and found that PLSR, by extracting principal components, can effectively handle high correlations between independent variables, thereby improving the accuracy of predictive models. By extracting principal components, the information of both independent and dependent variables is integrated, reducing the impact of multicollinearity and resulting in more reliable regression coefficient estimates. Additionally, the PLSR model not only allows for prediction but also offers interpretability. By extracting principal components, it can explain the relationship between independent and dependent variables, helping to understand the underlying structure and influencing factors within the data.

The choice of model is crucial during the analysis and modeling of plant samples. Considering various factors, the PLSR model may be a more efficient choice for plant samples with relatively stable growth conditions and clearly defined linear characteristics in the data. This is because, under stable growth conditions, the physiological indicators and growth data of plants often exhibit regular linear relationships. The PLSR model can effectively capture these linear patterns and accurately predict and analyze the plant’s growth status by fitting the linear relationship between the independent and dependent variables (Hensold et al., 2019). However, when plant growth is influenced by multiple complex factors, such as climate change, uneven soil fertility, and pest infestations, which result in significant nonlinear data characteristics, the BP and SVM models demonstrate greater potential.

### 4.3 Limitations and future research directions

This study provides valuable insights for future research by comparing the inversion accuracy of different models under feature selection. However, the morphological and physiological characteristics of maize plants vary significantly across different growth stages, and the applicability of the inversion model at various growth stages requires further validation. In addition, due to the influence of regional environmental conditions, plant growth characteristics, and local climatic factors, the transferability of the model exhibits a certain degree of uncertainty across different regions. To improve the generalization ability and reliability of the model, it is essential to thoroughly investigate the mechanisms through which these factors affect the model transfer process.

## 5 Conclusion

Based on multispectral UAV remote sensing imagery, optimal spectral variables were selected using RF. Models such as BP neural networks, SVM and PLSR were then applied to effectively estimate nitrogen content in the corn canopy. The study found that:

(1) During the multispectral remote sensing inversion of PNC using UAVs, redundancy exists in the information provided by different feature variables. By applying random forest for feature selection, redundant spectral information can be effectively removed, thus improving modeling efficiency. This process enhances the model’s predictive capability and optimization, ultimately improving the applicability of the inversion model.
(2) During the four growth stages, the VIs GI and NRI show a strong correlation with the nitrogen content in the corn canopy, indicating that the green and red light bands can effectively be used for the inversion of corn’s biophysical and biochemical parameters.
(3) During the tasseling stage, the spectral indices RED, NRI, GI, NR, NDREI, and RVI, selected based on RF, were used as input variables for the PLSR model. The RMSE was 0.13, MAE was 0.11, and R² reached 0.76, indicating the optimal inversion performance. These results demonstrate that the model excels in both accuracy and reliability, providing strong support for the accurate retrieval of relevant information.
(4) A comparative analysis of three models based on RF feature selection shows that the PLSR model consistently outperforms the BP and SVM models in inversion accuracy. Overall, the PLSR model exhibits the highest inversion accuracy, followed by the SVM model, with the BP model showing slightly lower accuracy.

## Data Availability

The raw data supporting the conclusions of this article will be made available by the authors, without undue reservation.
